# Influence of C-reactive protein on the pharmacokinetics of voriconazole in relation to the *CYP2C19* genotype: a population pharmacokinetics analysis

**DOI:** 10.3389/fphar.2024.1455721

**Published:** 2024-08-20

**Authors:** Jing Ling, Xuping Yang, Lulu Dong, Yan Jiang, Sulan Zou, Nan Hu

**Affiliations:** Department of Pharmacy, The First People’s Hospital of Changzhou/The Third Affiliated Hospital of Soochow University, Changzhou, Jiangsu, China

**Keywords:** voriconazole, C-reactive protein, *CYP2C19* genetic polymorphisms, population pharmacokinetics, nonlinear mixed-effects model

## Abstract

Voriconazole is a broad-spectrum triazole antifungal agent. A number of studies have revealed that the impact of C-reactive protein (CRP) on voriconazole pharmacokinetics was associated with the *CYP2C19* phenotype. However, the combined effects of *CYP2C19* genetic polymorphisms and inflammation on voriconazole pharmacokinetics have not been considered in previous population pharmacokinetic (PPK) studies, especially in the Chinese population. This study aimed to analyze the impact of inflammation on the pharmacokinetics of voriconazole in patients with different *CYP2C19* genotypes and optimize the dosage of administration. Data were obtained retrospectively from adult patients aged ≥16 years who received voriconazole for invasive fungal infections from October 2020 to June 2023. Plasma voriconazole levels were measured via high-performance liquid chromatography coupled with tandem mass spectrometry (HPLC-MS/MS). *CYP2C19* genotyping was performed using the fluorescence *in situ* hybridization method. A PPK model was developed using the nonlinear mixed-effect model (NONMEM). The final model was validated using bootstrap, visual predictive check (VPC), and normalized prediction distribution error (NPDE). The Monte Carlo simulation was applied to evaluate and optimize the dosing regimens. A total of 232 voriconazole steady-state trough concentrations from 167 patients were included. A one-compartment model with first order and elimination adequately described the data. The typical clearance (CL) and the volume of distribution (V) of voriconazole were 3.83 L/h and 134 L, respectively. The bioavailability was 96.5%. Covariate analysis indicated that the CL of voriconazole was substantially influenced by age, albumin, gender, CRP, and *CYP2C19* genetic variations. The V of voriconazole was significantly associated with body weight. An increase in the CRP concentration significantly decreased voriconazole CL in patients with the CYP2C19 normal metabolizer (NM) and intermediate metabolizer (IM), but it had no significant effect on patients with the CYP2C19 poor metabolizer (PM). The Monte Carlo simulation based on CRP levels indicated that patients with high CRP concentrations required a decreased dose to attain the therapeutic trough concentration and avoid adverse drug reactions in NM and IM patients. These results indicate that CRP affects the pharmacokinetics of voriconazole and is associated with the CYP2C19 phenotype. Clinicians dosing voriconazole should consider the patient’s CRP level, especially in CYP2C19 NMs and IMs.

## Introduction

Voriconazole is a broad-spectrum triazole antifungal agent that is extensively used in the treatment or prevention of invasive fungal disease caused by pathogens of *Candida* and *Aspergillus* species, as well as some emerging pathogens such as *Scedosporium apiospermum* and *Paecilomyces lilacinus* (Dolton and McLachlan, 2014). Voriconazole is rapidly absorbed after oral administration and exhibits nonlinear pharmacokinetics with disproportionate increases in plasma concentrations with increasing doses (Denning et al., 2002). Large inter-individual and intra-individual variations in concentrations have been observed in patients with invasive fungal disease treated according to the recommended dosage regimen (Wang et al., 2014). Numerous parameters, including liver diseases, inflammation, *CYP2C19* gene polymorphisms, and drug–drug interactions, have been found to partly contribute to the variability (Veringa et al., 2017; Yan et al., 2018; Tang et al., 2019; Zhang et al., 2021). Therapeutic drug monitoring for voriconazole is recommended for optimizing outcomes and reducing toxicity in clinical practice (Park et al., 2012). However, therapeutic drug monitoring can only adjust the dose after starting treatment and reaching a steady state. Identifying the factors that contribute to the high variability in voriconazole pharmacokinetics is important for adjusting the appropriate dosage with the least delay possible.

Voriconazole is metabolized by the cytochrome P450 enzymes, and CYP2C19 is the main metabolic enzyme. *CYP2C19* exhibits high genetic polymorphism, with the CYP2C19*2, CYP2C19*3, and CYP2C19*17 mutations being major factors, leading to variability in the metabolism (Li-Wan-Po et al., 2010; Lee et al., 2012). Based on the Clinical Pharmacogenetics Implementation Consortium (CPIC) Guidelines for CYP2C19 and voriconazole therapy (Moriyama et al., 2017), the patients could be classified as ultrarapid metabolizers (UMs, CYP2C19*17/*17), rapid metabolizers (RMs, CYP2C19*1/*17), normal metabolizers (NMs, CYP2C19*1/*1), intermediate metabolizers (IMs, CYP2C19*1/*2, CYP2C19*1/*3, and CYP2C19*2/*17), and poor metabolizers (PMs, CYP2C19*2/*2, CYP2C19*2/*3, and CYP2C19*3/*3). Several studies have shown that the *CYP2C19* gene polymorphism is associated with the pharmacokinetics, efficacy, and safety of voriconazole (Abidi et al., 2015; Favela-Mendoza et al., 2015; Ho et al., 2017; Li et al., 2017). It has been found that *CYP2C19* genotype-guided voriconazole dosing may improve the efficacy and safety of voriconazole (Hicks et al., 2020; Patel et al., 2020).

Inflammation is part of a complex biological response to harmful stimuli, such as pathogens, damaged cells, or irritants (Shah and Smith, 2015). *In vivo* and *in vitro* studies have shown that inflammation may induce the downregulation of CYP450 expression, which decreases the metabolism of the drug, resulting in increased drug concentration (Shah and Smith, 2015; Encalada et al., 2016; Stanke-Labesque et al., 2020). In recent years, numerous studies have investigated the relationship between inflammation and voriconazole exposure (Li et al., 2022). The results revealed that the inflammatory status, represented by C-reactive protein (CRP) and procalcitonin (PCT), can significantly affect the concentration of voriconazole (van Wanrooy et al., 2014; Encalada et al., 2015; Veringa et al., 2017; Cheng et al., 2020; Luo et al., 2021). Invasive fungal infections are often associated with inflammation, which increases the risk of voriconazole overexposure. Therefore, frequent monitoring of voriconazole trough concentrations is recommended during and following inflammation. Moreover, a meta-analysis revealed that the effect of inflammation appeared to be less important for patients with loss-of-function polymorphisms in *CYP2C19* (Bolcato et al., 2021). However, in contrast to the findings of the meta-analysis, Vreugdenhil et al. (2018) argued that the impact of inflammation in patients who use voriconazole could be even greater in poor metabolizers.

Population pharmacokinetics (PPK) provides a quantitative analysis of the variables affecting the pharmacokinetic parameters and is a common method for individualized drug administration. Numerous PPK studies on voriconazole have been conducted in recent years (Shi et al., 2019). The most commonly identified covariates were body weight, the *CYP2C19* genotype, liver function, CRP concentration, and concomitant medications. However, the combined effects of *CYP2C19* genetic polymorphisms and inflammation on voriconazole pharmacokinetics have not been considered in previous PPK studies.

Therefore, we aimed to further quantify the impact of inflammation on the pharmacokinetics of voriconazole in patients with different *CYP2C19* genotypes. A PPK model was constructed to identify the factors that significantly impact the pharmacokinetic parameters and provide dose optimization for the clinical rational use of voriconazole.

## Materials and methods

### Patients

This was a retrospective study performed at the Third Affiliated Hospital of Soochow University from March 2020 to August 2023. Inpatients aged ≥18 years with either a proven, probable, or possible diagnosis of invasive fungal infections treated with voriconazole and who underwent therapeutic drug monitoring were included. The exclusion criteria were as follows: 1) the absence of clinical data and 2) the combination of other antifungal agents or medications that significantly affect the pharmacokinetics of voriconazole. Demographic characteristics and clinical data were accurately extracted from the electronic medical records of each patient, including voriconazole treatment and therapeutic drug monitoring data and other factors that potentially influence the voriconazole trough concentration, such as CRP, albumin (ALB), alanine aminotransferase (ALT), aspartate transaminase (AST), total bilirubin (TBIL), hemoglobin (HB), platelet count (PLT), serum creatinine (Scr), and uric acid (UA), which were measured within the same day. The study was conducted in accordance with the guidelines of the Declaration of Helsinki and was approved by the Ethics Committee of the Third Affiliated Hospital of Soochow University (No. 2023-038).

### Blood sampling and analytical assays

For the patients receiving the loading dose, the initial plasma sample was collected on the third day of treatment. For the patients without a loading dose, the plasma samples were collected after 5 days of treatment. All samples were obtained within 30 min before the next administration. Blood samples were centrifuged within 6 h and stored at −80°C; plasma was used to detect the concentration of voriconazole, and white blood cells were used for CYP2C19 identification. Plasma voriconazole concentration was measured using high-performance liquid chromatography coupled with tandem mass spectrometry. The plasma samples (50 μL) were precipitated with methanol-containing isotope internal standards. The chromatographic separation was performed on a Kinetex C_18_ Column (3 mm × 100 mm, 2.6 μm) using a mobile phase of 0.1% formic acid–water (containing 5 mmol·L^–1^ of the ammonium acetate solution) and 0.1% formic acid–methanol at a flow rate of 0.6 mL·min^–1^. The injection volume was 5 μL, and the analysis time was 5 min. The detection of the analytes was performed via electrospray ionization in the positive mode by multiple reaction monitoring. The plasma concentration of voriconazole was linear over the range of 0.1–20 μg·mL^–1^. The intra- and inter-day precisions were 1.92% and 4.60%, respectively.

### Genotyping and phenotype assignment

CYP2C19 genotyping was performed by the fluorescence *in situ* hybridization method by using a fluorescence detector (Xi’an Tianlong Science and Technology Co., Ltd.). According to nomenclature by CPIC, the *CYP2C19* phenotypes were classified into five categories: UMs (CYP2C19*17/*17), RMs (CYP2C19*1/*17), NMs (CYP2C19*1/*1), IMs (CYP2C19*1/*2, CYP2C19*1/*3, and CYP2C19*2/*17), and PMs (CYP2C19*2/*2, CYP2C19*2/*3, and CYP2C19*3/*3).

### Population pharmacokinetic modeling

PPK analysis was conducted using the nonlinear mixed-effects model (NONMEM) program version 7.3 (ICON Development Solutions). The first-order conditional estimation method with interaction (FOCE-I) was selected to estimate the model. Graphical and statistical analyses were performed using R (version 2.15.1). A one-compartment model with first-order absorption and linear/non-linear elimination was used to analyze the pharmacokinetics of voriconazole following oral or intravenous administration. Given the small proportion (less than 5%) of the concentrations below the quantification limit (BQL), data were analyzed using the M1 methodology (discarding the BQL observations) (Xu et al., 2011). As the retrospective dataset consisted solely of trough concentrations and there were no data from the absorption phase, k_a_ was fixed at 1.1 h^–1^, as previously reported by Pascual et al. (2012). The inter-individual variability in PK parameters was described using an exponential model:

$$P_{j}=\hat{P}\times \exp\;\left(\eta_{j}\right),$$

where $P_{j}$ represents the pharmacokinetic parameter estimation for the *j*th individual, $\hat{P}$ represents the population typical value of the parameters, and $\eta_{j}$ is a random variable distributed with a mean of 0 and a variance of ω^2^. Residual variability was evaluated using the exponential and additive combined error model:

$$C_{ij}={\hat{C}}_{ij}\times \exp\left(\varepsilon_{1}\right)+\varepsilon_{2},$$

where $C_{ij}$ represents the *j*th observation for the *i*th patient ${\text{and}\;\hat{C}}_{ij}$ represents the *j*th predicted value for the *i*th patient. $\varepsilon_{1}$ and $\varepsilon_{2}$ are the intra-individual variabilities with a mean of 0 and variances of σ_1_^2^ and σ_2_^2^, respectively.

Covariate model exploration was conducted after the selection of the basic model. Age, gender, body weight, the *CYP2C19* phenotype, co-administered medications, liver and renal function indicators, and complete blood count were used as possible covariates to determine whether they explained the PK variability of voriconazole among patients. Pairwise correlations between all variables were assessed prior to covariate analysis, and highly collinear variables (correlation coefficient >0.5) would not be simultaneously incorporated into the final model. Continuous covariates were centered at their medians and explored using linear, power function, and exponential models. The categorical covariates were evaluated using the power model. The final model was built using stepwise covariate modeling with forward inclusion followed by a backward elimination method. It was considered significant when the inclusion of a covariate decreased the objective function value (OFV) by at least 3.84 (*p* < 0.05) and increased the OFV by at least 6.63 (*p* < 0.01) in the backward elimination.

### Model validation

Goodness-of-fit plots were constructed to evaluate the adequacy of fitting. A total of 1,000 bootstrap replicates were used to assess the robustness and stability of the final model. All the parameters (median values and 95% confidence intervals) obtained from the bootstrap were compared with the final model parameter estimates. Furthermore, the normalized prediction distribution error (NPDE) and visual predictive check (VPC) were performed to evaluate the predictive performance of the final model.

### Dosage regimen simulations

The Monte Carlo simulation was performed for each dosage regimen based on the final model. The target trough concentration range was defined as 0.5–5.0 μg/mL, which was recommended in the Chinese Practice Guidelines for the individualized medication of voriconazole (Chen et al., 2018). The probability of target attainment for the target trough concentration range was calculated for each of the different dosing regimens.

## Results

### Patient characteristics

One hundred and sixty-seven patients with a total of 232 voriconazole trough concentrations were included in the study. The demographics and clinical information of the patients are summarized in Table 1. Fifty-seven patients received an intravenous loading dose of 600 mg or 400 mg twice daily, with a subsequent intravenous maintenance dose of 300 or 200 mg twice daily; four patients received an oral loading dose of 400 mg or 300 mg twice daily and an oral maintenance dose of 200 mg twice daily; and one hundred and six patients were administered intravenously or orally at a dose of 200 mg twice daily without a loading dose. Oral preparations were taken 1 h before or after the meal. The rate of intravenous infusion was less than 3 mg/kg per hour. During subsequent treatment, the physician would adjust the dose according to the voriconazole concentrations. There was an extensive variation in the voriconazole trough concentration, with a median concentration of 4.45 μg/mL and a range of 0.25–16.75 μg/ml; 59.1% (137/232) of the trough concentration values were within the range of 0.5–5.0 μg/mL. Among the patients, there were 66 NM patients, 72 IM patients, and 29 PM patients. No UM or RM patients were included in the study. A total of 117 (70.1%) patients received a co-administration of proton pump inhibitors, and 43 (25.7%) patients received a co-administration of glucocorticoids.

**TABLE 1:** Demographics and clinical information of the study patients (*n* = 167).

Characteristic	Value^a^
Gender (M/F), n	119/48
Age (y)	68.87 ± 14.87 (71, 16–97)
Weight (kg)	64.48 ± 12.24 (65, 37–100)
Concentration (μg·mL^−1^)	4.67 ± 2.66 (4.45, 0.25–16.75)
Aspartate transferase (U·L^−1^)	63.98 ± 109.94 (34.7, 7.8–1211.6)
Alanine transferase (U·L^−1^)	44.42 ± 68.84 (25.35, 2.6–629.1)
Albumin (g·L^−1^)	36.36 ± 8.54 (34.8, 18.3–75.1)
Total bilirubin (μmol·L^−1^)	26.65 ± 46.64 (13.35, 1.7–514.8)
Hemoglobin (mmol·L^−1^)	97.96 ± 16.52 (96, 64–152)
Platelet count (×10^9^ L^−1^)	174.25 ± 114.66 (165, 4–624)
Serum creatinine (μmol·L^−1^)	106.50 ± 84.18 (78, 2.77–641)
Uric acid (μmol·L^−1^)	196.97 ± 163.86 (156.8, 27.4–1917)
C-reactive protein (mg·L^−1^)	77.10 ± 68.74 (58.85, 0.9–306.6)
Concomitant medication	n (%) of patients
Proton pump inhibitor	117 (70.1%)
Corticosteroid	43 (25.7%)
*CYP2C19* genotype Normal metabolizer	66 (39.5%)
Intermediate metabolizer	72 (43.1%)
Poor metabolizer	29 (17.4%)

^a^ The results for continuous covariates are presented as mean ± SD (median and range), and the results for categorical covariates are presented as frequency (percentage).

### Population pharmacokinetic analysis

A one-compartment model with first-order absorption and elimination was found to be the best base model due to the lowest OFV and the best goodness-of-fit plots. The model was parameterized using k_a_, clearance (CL), volume of distribution (V), and bioavailability (F). The assignment of inter-individual variability on F did not improve the data description and resulted in a large shrinkage (90.63%). As a result, inter-individual variabilities were detected for CL and V. The population pharmacokinetic parameters related to the base model are listed in Table 2.

**TABLE 2:** Pharmacokinetic parameters of voriconazole and bootstrap results.

Parameter	Base model	Final model	Bootstrap	
	Estimate (RSE%)	Estimate (RSE%)	Median	95% CI
*k*_a_ (h^−1^)	1.1 (fixed)	1.1 (fixed)	1.1 (fixed)	—
CL (L/h)	3.13 (5.9)	3.83 (9.5)	3.84	3.17∼4.77
V (L)	93.2 (32.8)	134 (12.0)	134	99.7∼169.9
F (%)	0.957 (9.3)	0.965 (7.4)	0.965	0.829∼1.110
CRP on CL		−0.155 (25.2)	−0.153	−0.273∼−0.076
IM on CL		0.794 (8.0)	0.783	0.657∼0.905
PM on CL		0.635 (12.0)	0.630	0.472∼0.781
ALB on CL		0.644 (26.4)	0.683	0.300∼1.100
Gender on CL		1.410 (7.2)	1.410	1.203∼1.619
Age on CL		−0.582 (25.3)	−0.602	−0.928∼−0.307
WT on V		2.210 (28.3)	2.215	0.705∼3.408
Inter-individual variability				
CL (%) (% shrinkage)	45.9 (23.6) (13.3)	38.9 (20.4) (16.2)	37.7	28.4∼46.7
V (%) (% shrinkage)	47.0 (53.4) (77.0)	45.2 (31.1) (65.7)	46.4	16.5∼72.2
Residual error				
Prop (%) (% shrinkage)	16.3 (29.8) (35.0)	14.7 (27.4) (35.3)	14.3	7.82∼18.6
Add (μg/mL) (% shrinkage)	0.78 (22.0) (35.0)	0.58 (30.2) (35.3)	0.55	0.23∼0.74

*k*_a_, absorption rate; CL, clearance; V, volume of distribution; F, oral bioavailability; Prop, proportional error; Add, additive error; RSE, relative standard error; CRP, C-reactive protein; IM, intermediate metabolizer; PM, poor metabolizer; ALB, albumin; WT, body weight; CI, confidence interval.

The stepwise covariate modeling procedure revealed that CL was significantly affected by CRP (ΔOFV = 26.47), the *CYP2C19* phenotype (ΔOFV = 21.26), albumin (ΔOFV = 20.14), gender (ΔOFV = 18.64), and age (ΔOFV = 16.31). Body weight (ΔOFV = 11.51) was found to significantly affect V. An increase in CRP levels significantly decreased the voriconazole CL in *CYP2C19* NM and IM patients. However, the inhibitory effect of CRP appeared to be less important in *CYP2C19* PM patients. Specifically, when CRP was tested in all *CYP2C19* phenotype patients, the fitted exponential factors were −0.215, −0.109, and −0.0172 in *CYP2C19* NM, IM, and PM patients, respectively. On the basis of these results, as the CRP concentration increased from 1 to 100 mg·L^−1^, the CL decreased by 34%, 19%, and 0.03% in *CYP2C19* NM, IM, and PM patients, respectively. Considering that CRP has little effect on PM patients, this factor was not included in the model. Moreover, the same fitting exponential factor was used to fit the model in both *CYP2C19* NM and IM patients. There was no significant increase in the OFV (ΔOFV = 0.994). Hence, for *CYP2C19* NM and IM patients, the same fitting exponential factor of CRP was used in the final model. The final model included the following equations: $CL\left(L/h\right)=3.83\times 0.794^{\left(IM=1\right)}\times 0.635^{\left(PM=1\right)}\times e^{\left(-0.155\times \frac{CRP}{59}\right)\times \left(NM=1、IM=1、PM=0\right)}\times {\left(\frac{AGE}{71}\right)}^{-0.582}\times {\left(\frac{ALB}{34.8}\right)}^{0.644}\times 1.41^{gender\left(M=0、F=1\right)}$ ; $V\left(L\right)=134\times {\left(\frac{WT}{65}\right)}^{2.21}$ ; *F* = 96.5%. The final model parameters are summarized in Table 2.

### Model validation

**FIGURE 1:**
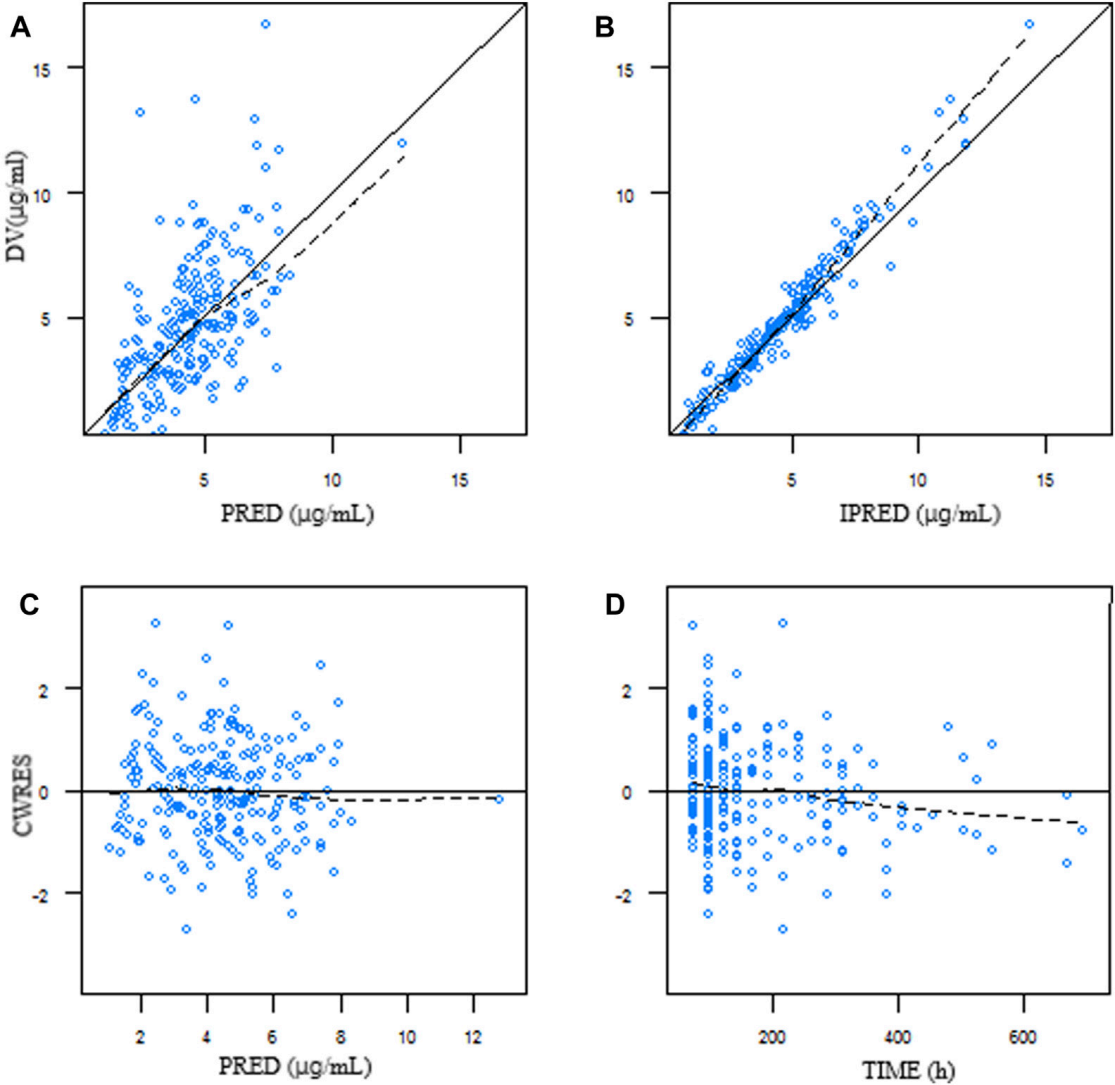
Goodness-of-fit plots of the final model. **(A)** DV plotted against PRED. **(B)** DV plotted against IPRED. **(C)** Correlation of CWRES with PRED. **(D)** Correlation of CWRES with the time after dosing. The line represents the line of identity. DV, observed concentration; PRED, predicted concentration; IPRED, individual predicted concentration; CWRES, conditional weighted residuals.

Goodness-of-fit plots from the final model illustrated that both the predicted concentration (PRED) and the individual predicted concentration (IPRED) corresponded well with the observed value (DV). Most of the conditional weighted residuals were located between ±2 (Figure 1).

The bootstrap (*n* = 1,000) results are summarized in Table 2. The results showed that 898 out of 1,000 runs converged successfully. The estimated values of the final model were similar to the median values from the bootstrap results and fell within 95% confidence intervals, suggesting good accuracy and stability of the final model.

**FIGURE 2:**
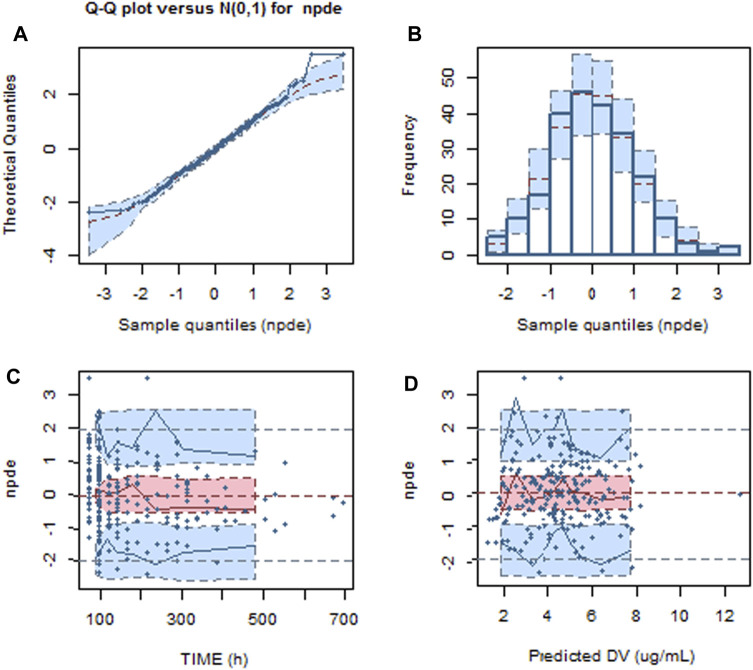
Normalized predictive distribution error (NPDE) of the final model. **(A)** Q–Q plot of the NPDE. **(B)** Histogram of the NPDE. **(C)** NPDE versus time after dosing. **(D)** NPDE versus population predicted concentration.

**FIGURE 3:**
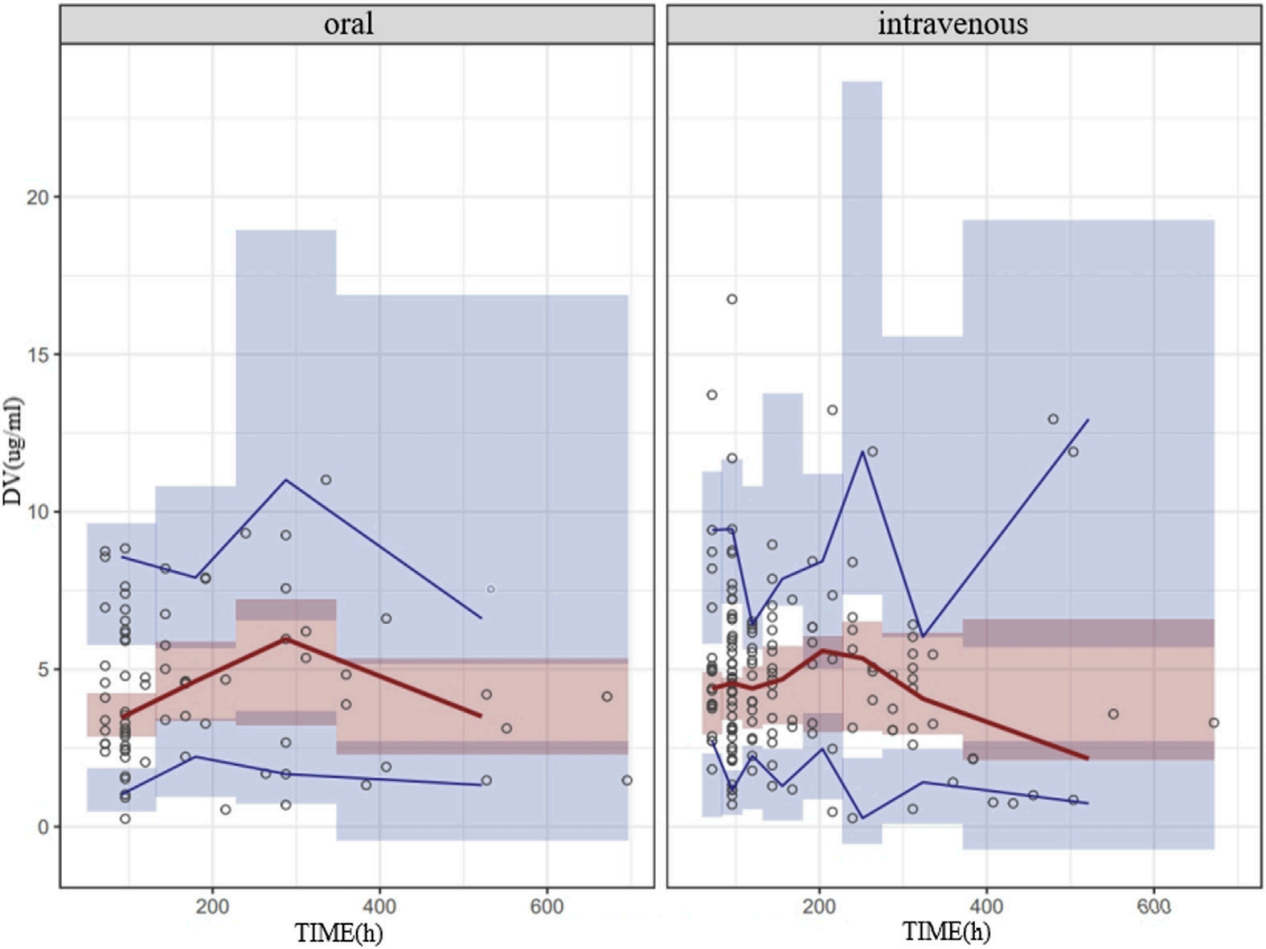
VPC of the final model for oral and intravenous administration of voriconazole. The hollow dots represent the observed data. Solid lines represent the 5th, 50th, and 95th percentiles of the observed data. Shaded areas represent the 95% confidence interval of the 5th, 50th, and 95th percentiles of the simulated data.

The NPDE result is presented in Figure 2. The mean and variance of the final model are 0.0257 and 1.06, respectively. The values of the *t*-test, Fisher test, and Shapiro–Wilk test of normality and global-adjusted *p*-value were 0.705, 0.490, 0.198, and 0.595, respectively. The results indicate that the final model has good predictive performance. The VPC plots displayed in Figure 3 showed that the model performed well in predicting the concentration of voriconazole for both oral and intravenous administration.

### Simulation

**FIGURE 4:**
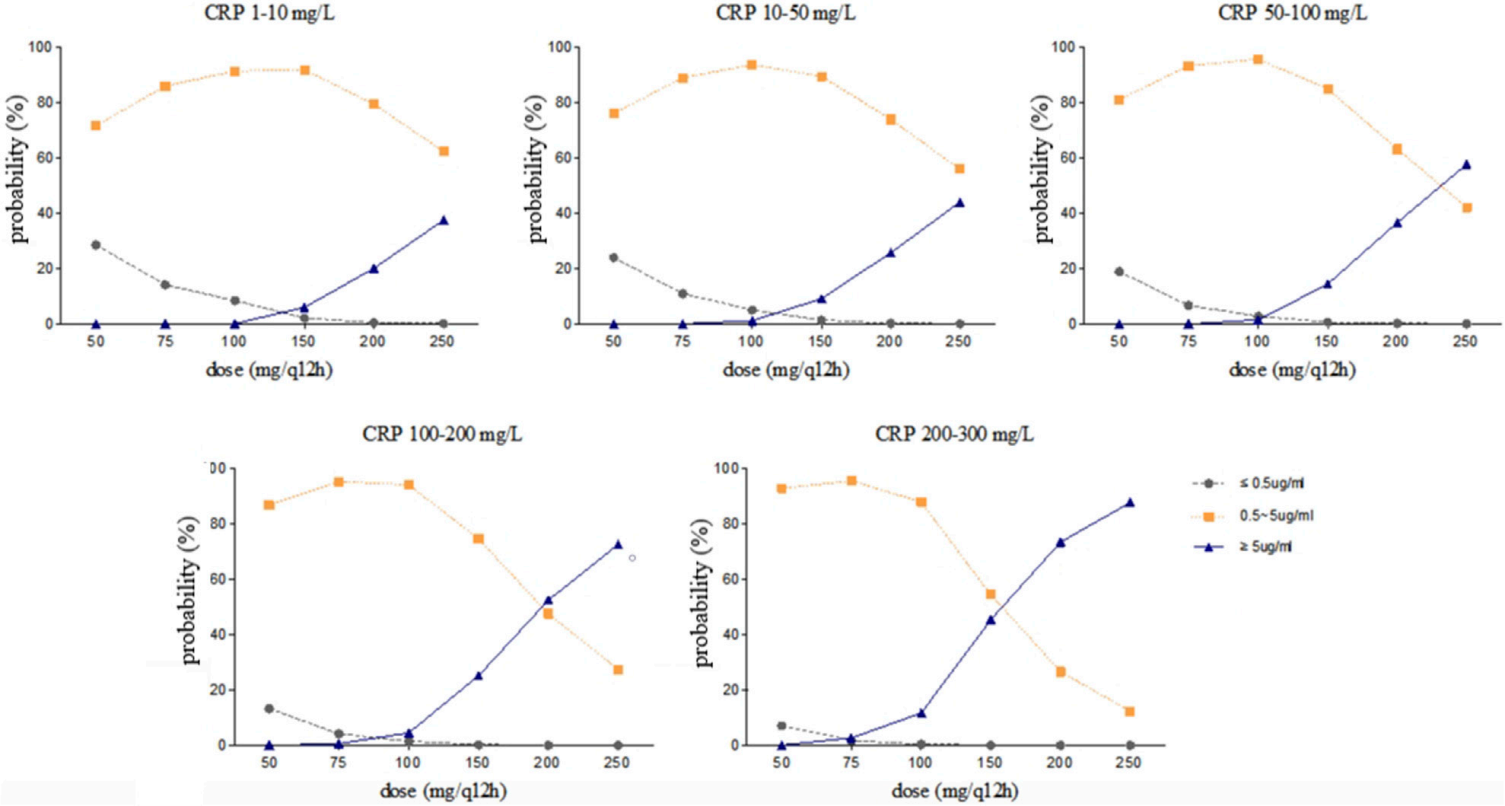
Simulated trough concentration stratified by CRP with the dosage regimens of 50, 75, 100, 150, 200, and 250 mg intravenous infusion twice daily in *CYP2C19* NM patients.

**FIGURE 5:**
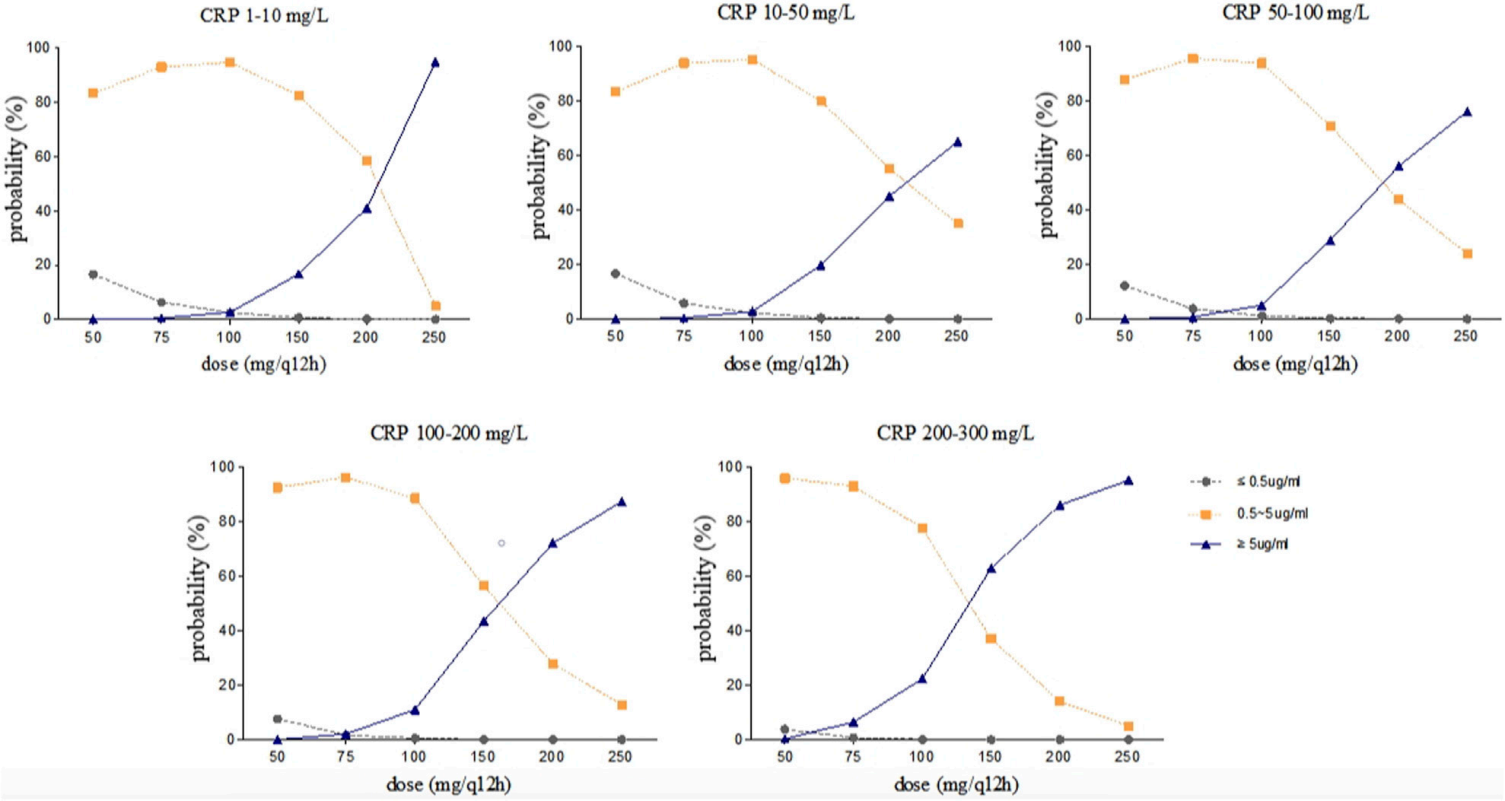
Simulated trough concentration stratified by CRP with the dosage regimens of 50, 75, 100, 150, 200, and 250 mg intravenous infusion twice daily in *CYP2C19* IM patients.

Steady-state trough concentrations of voriconazole were simulated based on the final model using the dosage regimens of 50, 75, 100, 150, 200, and 250 mg by intravenous infusion twice daily in typical patients (a 71 year-old-man with a body weight of 65 kg and an albumin concentration of 34.8 g/L) with various CRP and *CYP2C19* genotypes. The simulation trough concentrations are shown in Figures 4, 5. For example, in the group with CRP levels less than 10 mg/L, the 200 mg twice daily dosage regimen yielded therapeutic trough concentrations (0.5∼5.0 μg/mL) in 79.40% and 58.70% of *CYP2C19* NM and IM patients, respectively, whereas the percentages that reached the toxic range were 20.10% and 41.10%, respectively. However, in the group with CRP levels more than 200 mg/L, the percentage of trough concentrations that achieved the therapeutic range (0.5∼5.0 μg/mL) was 26.65% and 14.06% in *CYP2C19* NM and IM patients, respectively, whereas the percentages that reached the toxic range were 73.34% and 85.94%, respectively.

## Discussion

This retrospective study developed a population pharmacokinetic model of voriconazole in Chinese patients. To the authors’ knowledge, this is the first model-based study that focused on describing the influence of inflammation on the pharmacokinetics of voriconazole in relation to the *CYP2C19* genotype. A one-compartment linear elimination model with first-order absorption best described the data. Potential covariates affecting the voriconazole plasma concentration were obtained based on the model. The typical population values for CL, V, and F were 3.83 L/h, 134 L, and 96.5%, respectively. This is aligned with previously reported models (Chen et al., 2015; Lin et al., 2018; Ren et al., 2019; Jiang et al., 2022). The shrinkage values of CL and V are 16.2% and 65.7%, respectively. The large shrinkage of V may be due to the absence of the data in the distribution phase.

The impact of inflammation on voriconazole exposure is substantial. Severe infections and inflammation reactions commonly appeared in invasive fungal infection patients (Boglione-Kerrien et al., 2023). CRP is an acute-phase protein produced by hepatocytes and triggered by pro-inflammatory cytokines such as IL-6, which can reflect the severity of inflammation. As the CRP concentration is widely used as a marker for inflammation and is measured frequently in daily practice, CRP was chosen as a covariate in our model. In our study, the CRP concentration had a significant effect on voriconazole pharmacokinetics. These findings are comparable to those of previously reported studies, which show that CYP450 isoenzymes are decreased at the transcriptional levels during inflammation, leading to reduced metabolism of voriconazole (van Wanrooy et al., 2014; Encalada et al., 2015; Vreugdenhil et al., 2018; Li et al., 2022). Increases of 10, 50, 100, and 200 mg/L in CRP concentrations were associated with a decrease in the CL of voriconazole by 3, 15, 34, and 61%, respectively. This covariate is of the same order of magnitude as that found in a Chinese study (Jiang et al., 2022).

Moreover, our data confirmed that the impact of CRP on voriconazole pharmacokinetics was associated with the *CYP2C19* phenotypes. An increase in the CRP concentration significantly decreased the voriconazole CL in patients with *CYP2C19* NM and IM, but there were no significant effects on patients with *CYP2C19* PM. These results were consistent with those of a previous meta-analysis study, which demonstrated a smaller effect of inflammation for patients with decreased metabolic capacity than those with normal or elevated metabolic capacity (Bolcato et al., 2021). Although the results of the study by Chen et al. (2022), conducted in immunocompromised children <18 years of age, were inconsistent with our findings, their study showed that the impact of CRP on the trough concentration of voriconazole was modulated by the *CYP2C19* phenotype. The extent of the increase in the voriconazole trough concentration was greater in the IM group than in the PM and NM groups. The difference may be explained by age. Moreover, Gautier-Veyret et al. (2017) showed a greater effect of inflammation on voriconazole metabolism in ultrafast metabolizers. The conflicting results could be explained by the genetic factors that integrated both *CYP2C19* and *CYP3A* polymorphisms in their study. In addition, a higher frequency of PM of *CYP2C19* was observed in the Asian population than those in the Caucasian population (Shimizu et al., 2003). Hence, ethnicity differences cannot be ignored.

Seventy percent of patients received a combination of proton pump inhibitors. Proton pump inhibitors are metabolized by CYP2C19, CYP3A4, and CYP2C9, and they competitively inhibit the metabolism of voriconazole. The degree of influence varies with the type of proton pump inhibitors (Yan et al., 2018). In this study, no significant effect of proton pump inhibitors on voriconazole pharmacokinetics was observed. This may be related to the fact that most of the patients included in this study used rabeprazole or pantoprazole, while a few patients received omeprazole. However, the concentration of voriconazole should be closely monitored when combined with proton pump inhibitors.

The covariate age was shown to have a significant effect on the CL of voriconazole. In our study, the median age of the patients was 71 years, and a majority of the patients were elderly (aged ≥ 65). The association between the CL of voriconazole and age is consistent with the fact that voriconazole is metabolized by drug-metabolizing enzymes and with the known negative relationship between age and enzyme functional activity (Shi et al., 2019). This finding is similar to that of the study conducted by Wang et al. (2014) Therefore, it is especially necessary to monitor the voriconazole concentration in elderly patients.

Gender, albumin, and body weight were commonly identified variables in previous analyses (Lin et al., 2018; Ren et al., 2019; Allegra et al., 2020). In the present study, the CL of voriconazole was greater in women than in men, which is consistent with the results of Allegra et al. (2020). This phenomenon could be explained by differences in sexual hormones and body fat percentage (Allegra et al., 2020). The covariate albumin was shown to have a significant effect on the CL of voriconazole. Several studies have suggested that albumin is associated with the concentration of voriconazole (Vanstraelen et al., 2014; Khan-Asa et al., 2020; Takahashi et al., 2022). Therefore, toxic reactions should be closely monitored in patients with low albumin levels. Moreover, V, instead of CL, was increased with body weight. Hence, the dosage of voriconazole adjusted based on body weight in adults may not be feasible; instead, using ideal body weight or adjusted body weight might be more appropriate, according to a previous study (Koselke et al., 2012).

One of the main limitations to this study is its retrospective design. Only trough concentrations were obtained, which made it difficult to estimate the pharmacokinetic parameters in the absorption and distribution phases. In addition, there might be some deviation in the results due to the limited sample size. Furthermore, genetic polymorphisms of CYP3A4, ABCB1, and FMO3 that might influence voriconazole pharmacokinetics were not analyzed. Finally, the determination of the major metabolite voriconazole N-oxide may be useful in evaluating the variability in voriconazole concentration. Future studies with larger sample sizes and more comprehensive patient data are recommended to further investigate the outcomes.

## Conclusion

The population pharmacokinetic model developed in this study well described the plasma concentration of voriconazole. Dosing recommendations for voriconazole require considering the CRP level and *CYP2C19* phenotypes. When administering voriconazole in *CYP2C19* NM and IM patients with inflammation, close monitoring of the plasma concentration and proper adjustment of the dosage are recommended to maintain the voriconazole level within the therapeutic range and improve clinical outcomes.

## Data Availability

The original contributions presented in the study are included in the article/supplementary material; further inquiries can be directed to the corresponding author.
